# Overcoming anatomical prohibition: Cross-bar Nuss elevation as a gateway to minimally invasive mitral valve repair

**DOI:** 10.1016/j.xjtc.2026.102290

**Published:** 2026-02-23

**Authors:** Nunzio Davide de Manna, Alaa Selman, Khalil Aburahma, Fabio Ius, Bastian Schmack, Alexander Weymann, Arjang Ruhparwar, Patrik Zardo, Jawad Salman

**Affiliations:** Department of Cardiothoracic, Transplant and Vascular Surgery, Hannover Medical School, Hannover, Germany

**Figure:**
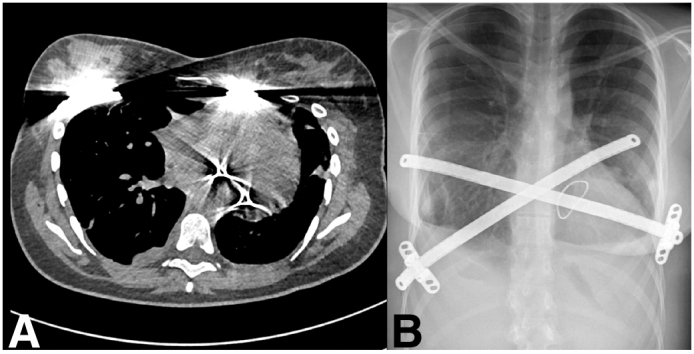
A, Postoperative CT findings: The modified cross-bar Nuss repair in Grand Canyon–type PE showed improvement in the sternal depression (Haller index, 2.06). B, Postoperative chest radiograph after cross-bar technique.

**Central Message**

Cross-bar sternal elevation reestablishes thoracic geometry and permits secure minimally invasive mitral repair in severe PE, enabling a true single-stage, single-recovery strategy.

Pectus excavatum is the most common anterior chest wall deformity and may lead to significant cardiopulmonary compromise from cardiac compression and displacement. Severe pectus excavatum can exacerbate right ventricular dysfunction and is frequently associated with mitral valve pathology, including mitral valve prolapse. Although the Nuss procedure is well established^1^ for minimally invasive pectus excavatum repair, its use alongside mitral valve surgery in a true single-stage, fully minimally invasive approach is rarely reported. We describe a combined modified cross-bar Nuss repair and minimally invasive mitral valve repair performed in a single operation to optimize exposure and restore thoracic geometry.

## Case Presentation

**Figure 1:**
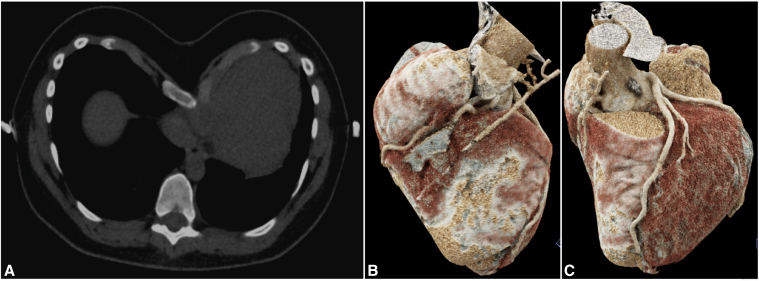
A, Preoperative axial computed tomography scan demonstrating marked cardiac displacement toward the left hemithorax due to severe pectus excavatum. B and C, Coronary computed tomography angiography showing normal coronary anatomy with no evidence of obstructive coronary artery disease or congenital anomalies.

A 22-year-old woman presented with progressive exertional dyspnea and intermittent chest pain. Transthoracic echocardiography revealed severe bileaflet mitral regurgitation (A2-P2 prolapse) with mildly reduced left ventricular ejection fraction. Cardiac computed tomography excluded coronary or congenital abnormalities (Figure 1, *B* and *C*) but demonstrated a severe “Grand Canyon–type’’ pectus excavatum (PE), an extreme morphologic variant in the Park classification characterized by a deep, narrow sternal depression with marked cardiac displacement (Haller index 4.5, Figure 1, *A*). Given the hemodynamic impact and anatomic distortion, a multidisciplinary team recommended a single-stage minimally invasive correction of both conditions. Written informed consent was obtained from the patient for publication of this case report and any accompanying images, in accordance with Committee on Publication Ethics guidelines.

**Figure 2:**
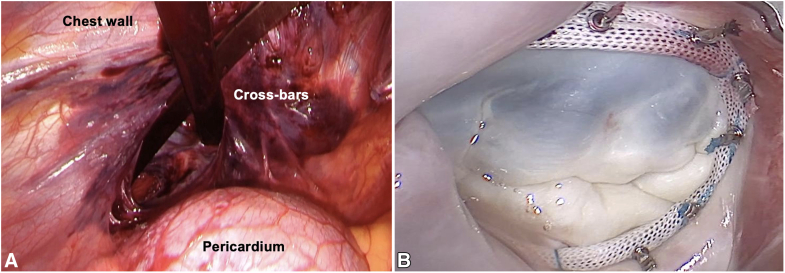
A, Internal thoracoscopic view showing the final configuration of the cross-bar Nuss repair with intersecting substernal bars in position. B, Intraoperative endoscopic mitral valve reconstruction

Under general anesthesia and selective lung ventilation, a modified cross-bar Nuss repair was performed first (requiring ∼30 minutes). Through 2 small lateral incisions, thoracoscopic guidance enabled placement of 2 prebent titanium bars (MedXpert GmbH) in a crossed configuration, positioned to preserve unobstructed right thoracic access, providing immediate and substantial sternal elevation (Figure 2, *A*).

The bars were initially left unsecured to allow access flexibility and rapid removal if sternotomy was required and definitively fixed after decannulation and protamine.

Thoracoscopic intercostal cryoanalgesia was performed using the cryoICE system (AtriCure) to optimize perioperative pain control. The bars were secured with stabilizers and high-strength sutures. Intraoperative transesophageal echocardiography confirmed relief of right ventricular compression and restoration of mediastinal geometry.

**Figure 3:**
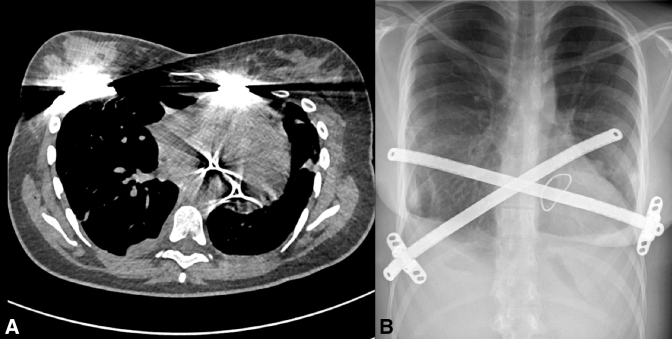
A, Postoperative computed tomography findings: The modified cross-bar Nuss repair in Grand Canyon–type PE showed improvement in the sternal depression (Haller index, 2.06). B, Postoperative chest radiograph after cross-bar technique.

Subsequently, peripheral cardiopulmonary bypass was initiated via the right femoral vessels, and a 4-cm right minithoracotomy afforded direct access to the left atrium. Mitral valve repair consisted of a single expanded polytetrafluoroethylene neochord to P2 and implantation of a 38-mm Physio II annuloplasty ring (Edwards Lifesciences), yielding symmetric coaptation with no residual regurgitation on saline testing (Figure 2, *B*). Postbypass transesophageal echocardiography confirmed a competent valve and normal gradients. Postoperative computed tomography confirmed complete correction of the anterior chest wall depression (Haller index, 2.06; Figure 3, *A*).

The patient had an uncomplicated recovery and was discharged on postoperative day 10. At the 3-month follow-up, echocardiography demonstrated durable repair, and she reported complete resolution of symptoms with excellent cosmetic and radiologic results (Figure 3, *B*).

## Discussion

Controversy remains regarding the concomitant repair of PE and mitral valve disease, particularly when both are approached through minimally invasive techniques. The pectus deformity itself may severely limit operative exposure of the mitral valve, whereas cardiopulmonary bypass–related coagulopathy increases the risk of bleeding after chest wall reconstruction. Balancing these competing risks requires precise timing, coordination, and technical control. Successful concomitant repair must fulfill several critical requirements: (1) minimized bleeding during pectus correction to prevent postoperative hemothorax under anticoagulated conditions; (2) optimal operative exposure for the intracardiac procedure, ensuring safe cannulation, cardioplegia delivery, and valve visualization despite altered thoracic anatomy; (3) stable yet flexible chest-wall fixation, allowing urgent postoperative reentry or effective cardiopulmonary resuscitation if required; and (4) acceptable cosmetic and functional outcomes, consistent with the principles of minimally invasive surgery.

In addition, effective perioperative analgesia is essential in this setting. Thoracoscopic intercostal nerve cryoanalgesia, performed before bar placement, has emerged as a valuable adjunct during minimally invasive repair of PE, significantly reducing acute postoperative pain and opioid requirements, and facilitating early mobilization—an important consideration when chest wall stability and respiratory mechanics are critical.^2^

In our case, performing the modified cross-bar Nuss correction first effectively resolved the issue of limited cardiac exposure by restoring normal thoracic geometry and relieving right ventricular compression. This modification allowed subsequent minimally invasive mitral valve repair via right minithoracotomy without the need for conversion to sternotomy. Of note, hemostasis was achieved before initiating cardiopulmonary bypass, minimizing bleeding risk.

Prior reports have described combined open or staged approaches for PE correction and cardiac surgery.^3^ However, only a few describe a true single-stage, minimally invasive technique,^4^ and none to our knowledge report the modified cross-bar configuration as a means of achieving safe cardiac exposure in an extreme “Grand Canyon” deformity.^5^

In selected cases of severe and complex PE, cross-bar configurations have been shown to provide superior sternal elevation and chest wall stability compared with parallel-bar techniques.E1, E2, E3

## Conclusions

The single-stage strategy offers the advantages of 1 anesthetic exposure, streamlined recovery, and high cosmetic satisfaction, but remains technically demanding and appropriate only for centers with integrated thoracic–cardiac expertise, particularly when optimal functional and cosmetic outcomes are sought in young patients. Although isolated reports describe sequential or partially combined approaches,^E4^ true concomitant minimally invasive repair is exceedingly rare. To our knowledge, this is the first case to integrate a modified cross-bar Nuss repair with minimally invasive mitral valve repair in a fully single-stage, single-recovery procedure, demonstrating both feasibility and favorable early outcomes.

### Declaration of Generative AI and AI-Assisted Technologies in the Writing Process

The authors used ChatGPT (OpenAI) to assist with language refinement and organizational clarity during manuscript preparation. All generated text was reviewed, verified, and edited by the authors, who take full responsibility for the content of the final manuscript.

## Conflict of Interest Statement

The authors reported no conflicts of interest.

The *Journal* policy requires editors and reviewers to disclose conflicts of interest and to decline handling or reviewing manuscripts for which they may have a conflict of interest. The editors and reviewers of this article have no conflicts of interest.

## References

[1] Salomon J., Shah P.M., Heinle R.A. (1975). Thoracic skeletal abnormalities in idiopathic mitral valve prolapse. Am J Cardiol.

[2] Bastianello M., Torre M., Bonfiglio R. (2025). Cryoanalgesia for pain management after pectus excavatum repair (COPPER) in adolescents: a randomized controlled trial. Paediatr Anaesth.

[3] Ishikawa N., Watanabe G., Horikawa T. (2021). Combined robot-assisted mitral valve plasty and Nuss procedure via small ports. Artif Organs.

[4] Khairallah S., Chow O.S., Mick S.L. (2022 Dec). Combined minimally invasive repair of pectus excavatum and robotically assisted mitral valve repair: a case report and considerations. J Card Surg.

[5] Haecker F.M., Krebs T.F., Kleitsch K.U. (2022). To cross or not to cross: the cross-bar technique to correct pectus excavatum with “Costal Flaring”.. Ann Thorac Surg Short Rep.

